# The Missing Link

**Published:** 1883-04

**Authors:** 


"THE MISSING LINK."—Whether the remarkable specimen of humanity now exhibited at the Westminster Aquarium is a "missing link" or not, nobody will deny that it is of the greatest interest. It is a child seven years of age, capable of speech, whose body is covered with short, dense, soft hair, with prehensile feet, hands capable of being bent back upon the wrist, pouched cheeks, used to store food as in the monkeys, and jaws slightly prognathic. It will not do nowadays to settle such an anomaly by calling it a *lusus naturæ*, for most naturalists are agreed that "sports" and "monstrosities" are often only "reversions to ancestral conditions." The father of the child was covered with hair in a similar manner. The family was discovered in the Lao country, by Mr. Carl Bock, the well-known traveler and anthropologist. Miss Bird, in her charming book on Japan, describes the short, hairy aboriginal race of that country known as Ainos; and more recently Mr. A. H. Keene has described the Aino ethnology more fully and scientifically. In the extreme east of Asia we have these dwarf hairy Ainos, fast becoming extinct; and now a Siamese hairy family turns up with decidedly Simian characteristics. In Burmah hairy people have been occasionally known. It will be remembered that, many years ago, Mr. Everett was sent to Borneo to explore the caves for possible early remains of man, as it has always been imagined that it is to the tropical and sub-tropical parts of the extreme East we should look for "missing links" between humanity and the lower animals.—*Science Gossip.*

A photograph of this famous "missing link," now lying before us (and for which we are indebted to Dr. J. S. Crapper of Hanley, England), shows it to be unmistakably human, with normal hands and feet, regular features of the African type, and jaws not at all prognathic. The only observable abnormality is a thin coat of hair which covers the entire body. The English people are evidently being Barnumized.—EDITOR.

